# Individual variation in feeding morphology, not diet, can facilitate the success of generalist species in urban ecosystems

**DOI:** 10.1002/ece3.8425

**Published:** 2021-12-07

**Authors:** Piatã Marques, Eugenia Zandonà, Rosana Mazzoni, Rana El‐Sabaawi

**Affiliations:** ^1^ Biology Department University of Victoria Victoria British Columbia Canada; ^2^ Departamento de Ecologia Universidade do Estado do Rio de Janeiro Rio de Janeiro Brasil

**Keywords:** adaptation, density‐mediated effects, intraspecific competition, urban ecology, urban species

## Abstract

Generalist species dominate urban ecosystems. The success of urban generalists is often related to a plastic diet and feeding traits that allow them to take advantage of a variety of food resources provided by humans in cities. The classification of a species as a generalist is commonly based on mean estimates of diet‐ and feeding‐related traits. However, there is increasing evidence that a generalist population can consist of individual specialists. In such cases, estimates based on mean can hide important individual variation that can explain trophic ecology and the success of urban dwellers. Here, we focus on guppies, *Poecilia reticulata*, a widespread alien fish species which has invaded both urban and non‐urban systems, to explore the effect of urbanization on individual diet and feeding morphology (cranium shape). Our results show that guppies in urban and non‐urban populations are not individual specialists, having a similar generalist diet despite the high population density. However, there is important individual variation in cranium shape which allow urban guppies to feed more efficiently on highly nutritious food. Our data suggest that individual variation in feeding efficiency can be a critical overlooked trait that facilitates the success of urban generalists.

## Graphical abstract

The success generalist species in urban systems is often related to a plastic diet and feeding‐related traits that allow them to take advantage of a variety of food subsidies provided by humans in cities. The classification of a species as generalist is commonly based on mean estimates of diet and feeding traits. However, there is increasing evidence that a generalist population can consist of individual specialists. Here, we show that individual variation in feeding morphology (head shape) can be important for facilitating the success of urban generalists.

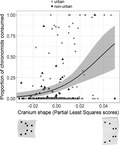

## 1 INTRODUCTION

The 21st century is undergoing intense urban land expansion and by 2030, 65% of all land area in the world is expected to become urban (D’Amour et al., 2017; Seto et al., 2012). Urbanization converts the land into a built landscape (D’Amour et al., 2017; Mcdonald et al., 2008) causing habitat loss, altering food, and water subsidies (Alberti, 2008). Despite the altered conditions, some species known as urban dwellers persist, reaching high densities in cities (Fischer et al., 2015).

Urban dwellers are most often generalist species that tolerate a wide range of habitat conditions and feeding conditions. This is often related to changes in characteristics (i.e., traits) that allow them to take advantage of the altered conditions imposed by urbanization (Alberti, Correa, et al., 2017). Increasing evidence suggests that changes in feeding traits are essential for persisting in cities (Alberti et al., 2017b). This is because they allow urban dwellers to adjust their diets to feed on a variety of food resources provided by humans (compost and sewage). Specifically, changes in head morphology seem important for the success of urban dwellers. For example, urban Red foxes and Anolis lizards have a skull morphology that accommodates stronger bite force which likely provides mechanical advantage for feeding on human‐derived food sources (Parsons et al., 1928; Winchell et al., 2016).

Evidence suggesting urban dwellers are generalist feeders come from studies that use the mean diet of all individuals in a population (Callaghan et al., 2019; Franzén et al., 1928). However, considerable variation can exist within a population, even among individuals of the same sex, age, and size (Bolnick et al., 2002). Such individual variation is often related to differences in behavior and morphology that affect feeding (Bolnick et al., 2003; Ferry‐Graham et al., 2002). As a result, individuals within populations can differ in such a way that a generalist population can be composed of many individual specialists that feed differently from the mean diet of the population (Pagani‐Núñez et al., 2015; Vander Zanden et al., 2010).

Individual specialization is mainly determined by intraspecific competition (Araújo et al., 2011). Individuals tend to avoid competition within a population by exploring alternative/unique food resources (Araújo et al., 2011; Bolnick et al., 2003; Roughgarden, 1974). The strength of competition is related to the accessibility of individuals to food, which is determined by food availability and population density that can be regulated by predation and competition (Araújo et al., 2011; Ferry‐Graham et al., 2002). Such mechanistic understanding comes from studies in non‐urban systems and the effect of urbanization on individual specialization remains fairly unknown.

**FIGURE 1:**
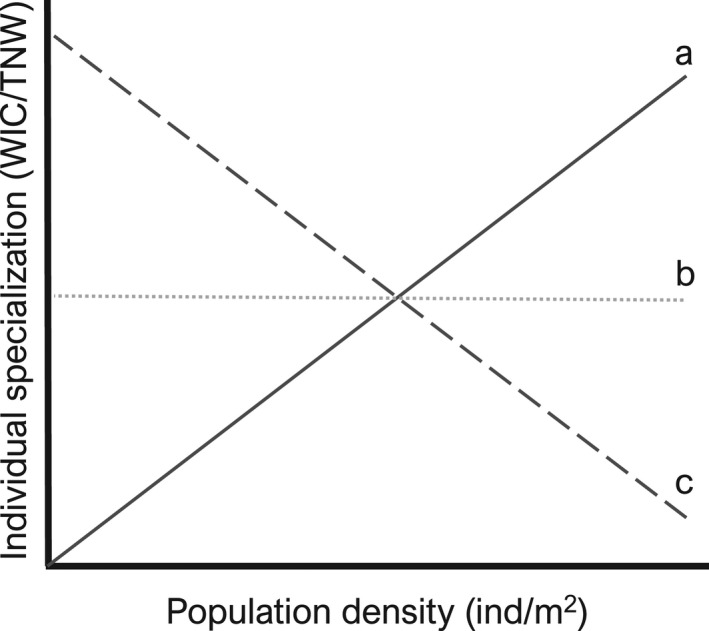
Possible effects of urbanization on individual specialization. Theory developed in non‐urban systems predict that because food is limited, increased population density enhances intraspecific competition, leading individuals to use alternative food sources which increase individual specialization (a). However, human subsidies increase food availability in urban systems. This can lead the emergence of new relationships between population density and individual specialization (b, c)

Urbanization can potentially disrupt the association between food availability and population density that defines individual specialization. Urban dwellers attain extremely high densities in urban areas, which is often related to high food availability (Møller et al., 2012; Šálek et al., 2015). Increased food availability in urban areas can be the result of a high supply of nutrients for primary production (e.g., ammonium and phosphate) and/or continuous supply of human‐provided food subsidies (e.g., animal feeders, compost, and garbage) (Alberti, 2008; Becker et al., 2018). Therefore, for urban dwellers, an increase in density may not necessarily correlate with increased resource competition or decline in per‐capita resource availability that enhances individual specialization (Figure 1). Understanding the factors shaping individual specialization and related feeding morphology can be fundamental to clarify the trophic mechanisms facilitating the dominance of generalist species in cities.

Here, we explore the effect of urbanization on individual specialization and feeding morphology using guppies, *Poecilia reticulata*. Guppies are native to the north of South America and the Caribbean, on the islands of Trinidad and Tobago (Magurran, 2005), but they have invaded many urban and non‐urban ecosystems across the globe (Araújo et al., 2009; Deacon et al., 2011; Lindholm et al., 2005). Guppies are omnivorous generalists with a tendency to feed on chironomids (midge larvae; Zandonà et al., 2011). Guppies have a larger body size, high fecundity, and population density in urban streams compared to non‐urban streams (Marques et al., 2020). Such success has been related to the increased mean consumption of chironomids, which dominate insect biomass in urban streams (Marques et al., 2020; Moreyra & Padovesi‐Fonseca, 2015). However, it is unclear whether the dietary difference between urban and non‐urban guppy populations is related to differences in density that sharpen individual specialization, or an opportunistic response to the increased availability of chironomids.

We begin by assessing how urbanization affects individual specialization in guppies. In non‐urban populations in their native range (i.e., Trinidad), high guppy population density is thought to increase competition with conspecifics by reducing food availability per capita (Grether et al., 2001; Reznick et al., 2001). High competition with conspecifics is expected to increase individual specialization (Svanback & Bolnick, 2007; Figure 1a). However, urban streams often have a high density of guppies at the same time that food availability is high (Cunico et al., 2006; Ganassin et al., 2019). This can confound the effect of density on competition which determines intraspecific specialization. If the effect of density on intraspecific specialization is similar between urban and non‐urban systems, we predict guppies to have increased individual specialization in urban streams (Figure 1a). On the other hand, if urbanization relax the density effects on individual specialization because of the increased food availability, we expect either no change in individual specialization or a decrease in urban streams (Figure 1b,c).

Then, we evaluate how individual specialization relates to trophic morphology. In native non‐urban populations, cranium morphology is related to diet. Guppies with shorter and wider cranium attain larger gape sizes, being more efficient to feed on chironomids (Palkovacs et al., 2011). Previous studies show that urbanization increases the mean consumption of chironomids by guppies (Ganassin et al., 2019; Marques et al., 2020). Thus, we predict urbanization to sharpen the traits (shorter and wider cranium) that increase foraging on chironomids.

## 2 MATERIAL AND METHODS

We used a study system in Rio de Janeiro, Brazil, described in Marques et al. (2020). In short, we have selected six urban and six non‐urban stream reaches in Rio de Janeiro, where guppies are known to occur. A map with the location of each sampled reach can be found in Marques et al. (2020). Urban stream reaches are characterized by having high concentration of fecal coliforms and ammonium, indicating they are contaminated with sewage. Within each treatment (urban and non‐urban), we had three reaches where guppies were the only fish (guppy only) and three reaches where guppies co‐occurred with fishes that are potential competitors and predators (guppy + other fish). In the latter reaches, the total number of species ranged from 3 to 10, and each reach had at least one potential predator, the catfish *Rhamdia quelen*, and one potential omnivorous competitor such as the poecilid *Phalloceros* sp. or the pearl cichlid *Geophagus brasiliensis* (Marques et al., 2020). Guppy density and food availability, particularly the biomass of midges (family Chironomid), was higher in urban than non‐urban stream reaches (~26× and ~3× higher, respectively; Marques et al., 2020) (Table A1 in Appendix). Guppies are larger in urban than in non‐urban stream reaches (Marques et al., 2020). Whenever possible we replicated the sampling in 2 years (2016–2017).

### 2.1 Guppy diet

**FIGURE A1:**
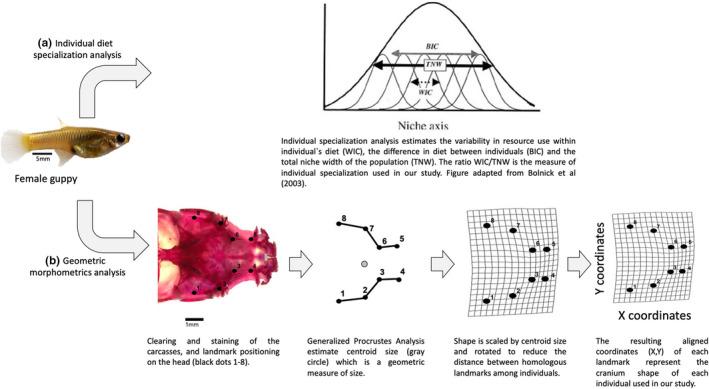
Analytical procedures. For this study, we used individual female guppies that were simultaneously assessed for individual diet specialization (a) and head morphology (b). Panel A shows the individual specialization metrics defined by Bolnick et al. (2003, 2002). Panels in (b) show the step‐by‐step procedures for analyzing morphology using the geometric morphometrics method following Zelditch et al. (2012)

In previous work, we show that urban and non‐urban guppies have different diet using estimates based on the mean food consumption (Marques et al., 2020). Now, to explore the source of mean dietary difference, we reanalyzed the same individuals previously used, focusing on individual variation. A total of 113 urban (10 ± 1 per reach) and 118 non‐urban (13 ± 1 per reach) female guppies were euthanized and fixed in formalin (10%) for further analyses (Figure A1 in Appendix). Guppies were collected and euthanized following protocols approved by the University of Victoria (2016‐008) and the State University of Rio de Janeiro (UERJ CEUA/005/2016) animal care committees, as well as the Brazilian Ministry for the Environment (IBAMA 16152‐1). We used only sexually mature female guppies to remove any diet variation related to ontogeny and sex. Each guppy was measured for standard length (mm, SL), dissected, and the foregut was sectioned to the point where the gut turns 180° (Zandonà et al., 2011). A detailed description of the gut content analysis can be found in Marques et al. (2020). Briefly, gut content analysis was performed using a gridded microscope slide (Zandonà et al., 2011). The slide area occupied by detritus (i.e., silt and amorphous material), algae, and invertebrates was estimated (mm^2^). We identified the invertebrates to the lowest taxonomic level possible, generally family, using published taxonomic keys (Merritt et al., 1996; Mugnai et al., 2010), while the algae were categorized into three broad groups: diatoms, filamentous, and others. We then used the total area of the slide to estimate the proportion of each food item. Because chironomids are especially important food for guppies, we separated the proportion of chironomids consumed (PC) from other invertebrates to use in further analyses.

### 2.2 Individual specialization estimates

We assessed the individual diet at each reach and year by considering the amount (area, mm^2^) of each invertebrate family, algae group (diatom, filamentous, and others), and the bulk of detritus consumed by each guppy. We used broad categories for algae and detritus because it is unlikely that guppies can distinguish algal families within groups or detritus type. We used individual specialization metrics to assess individual diet variation (Bolnick et al., 2002, 2003; Roughgarden, 1972, 1974) (details in Figure A1a in Appendix). Individual specialization was estimated for each stream reach and indicated how similar each individual's diet is compared to the diet of the whole population in the same reach. Through this approach, the total niche width of the population (TNW) is determined by the sum of the variability in resource use within individual's diet (WIC) plus the variability in diet between individuals (BIC). The ratio WIC/TNW is a measure of the degree of individual specialization in a population. Where, WIC/TNW values are closer to 0 indicate that the population is composed of individuals with narrow niche width, thus high individual specialization; while values close to 1 indicate individuals have wide niche width, thus high individual generalization (Bolnick et al., 2003). Individual specialization metrics were estimated using the function “WTcMC” of the *RInSp* package for R (Zaccarelli et al., 2013). This function also applies a Monte‐Carlo resampling technique to estimate p‐values testing the null hypothesis that the population is composed of generalist individuals who sample randomly from the population's diet distribution (Zaccarelli et al., 2013). Estimates were corrected so that all individuals have equal weight for calculating TNW, regardless of the number of items in the diet. Differences in WIC, BIC, TNW, and WIC/TNW were assessed using linear models with origin (urban vs. non‐urban), body length, and year as fixed factors and reach identity as a random factor.

### 2.3 Trophic morphology

**FIGURE 2:**
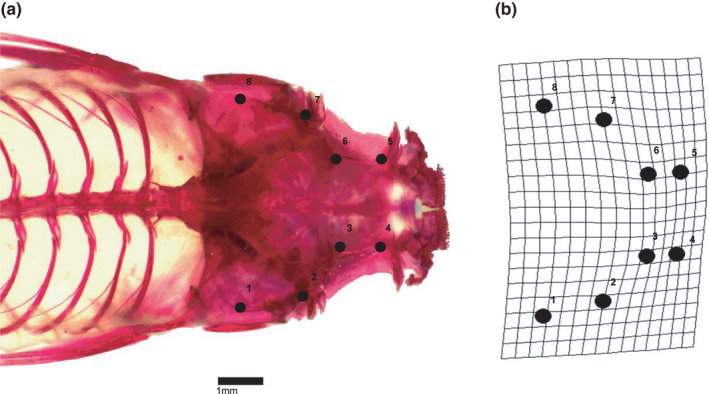
Analysis of the cranium shape of guppies. The panel (a) shows an image from the antero‐dorsal view of a female guppy. The bones are stained in red and the numbers show the landmarks used to define the cranium shape. The anatomical loci of each landmark are described as: 1 and 8 = the edge of the pterotic bone, 2 and 7 = posterior region of the sphenotic process, 3 and 6 = the crest of the frontal‐parietal bone, and 4 and 5 = the intersection between supraorbital part of the frontal bone and the base of the lachrymal bone. The panel (b) shows a thin‐plate spline deformation grid that represents the variation in shape of the individual in panel (a), in relation to the mean of all the individuals in the population. The deformation grid is based on the procrustes shape coordinates obtained after a generalized procrustes analysis using the shape landmarks shown in panel (a)

Following diet analysis, guppies were assessed for trophic morphology (cranium shape) (Figure 2). We used the same individuals analyzed for the diet except for 29 guppies with damaged carcasses. A total of 202 guppies (93 urban and 109 non‐urban) were assessed for trophic morphology. We gutted each guppy and passed the carcasses through a series of chemical solutions that cleared skin and muscles, and stained the bones red, following a protocol modified from Taylor & Van Dyke (1985) and Song & Parenti (1995). Each guppy was photographed (SPOT Imaging, Diagnostic Instruments, Inc.) after clearing and staining under a dissecting microscope (Wild Leica – M420, Leica Biosystems). The microscope magnification (10×) and guppy position on the field of view were kept constant to minimize individual images’ variation. Individual images were then analyzed for cranium shape, following a geometric morphometrics approach (Zelditch et al., 2012; details Figure A1b in Appendix). Such approach has advantages over traditional morphological analyses because it disentangles shape variables from size (detailed below). Images were uploaded into the software tps. Dig version 2.17 for digitalizing the cranium landmarks necessary for the shape analysis (Rohlf, 2013; Figure 2a). Landmarks are homologous anatomical loci among all individuals and provide an adequate description of the shape (Zelditch et al., 2012). We determined eight landmarks on the dorsal plane of the cranium, which together describe the cranium shape (modified from Palkovacs et al. [2011]; Figure 2a). The description of the anatomical location of each landmark was based on (Hernandez et al., 2008). The position (x, y coordinate) of each landmark for each individual was uploaded into R software. We used the function “gpagen” of the *geomorph* package for R to perform a generalized procrustes analysis of shape (GPA; Adams & Otárola‐Castillo, 2013). This procedure superimposes each individual (i.e., a set of landmarks) onto each other by centering, scaling, and rotating them (Zelditch et al., 2012). Centering subtracts the coordinate of the centroid (i.e., the distance of all landmarks of one individual to the center of the form, this is the measure of body size in geometric morphometrics) from the corresponding X and Y coordinates of each landmark. At the same time, the scaling procedure divides the X and Y coordinate of each landmark by the centroid size of that individual. The rotating process then spins each individual to reduce the distance between homologous landmarks. The resulting aligned coordinates of each landmark (procrustes shape variables) represent the cranium shape of each individual without the effect of body size which is stored as centroid size (Zelditch et al., 2012) (details Figure A1b in Appendix). Following GPA, 16 vectors of shape (X and Y coordinates for each one of the 8 landmarks that describe cranium shape) plus one vector that describe the geometric size of each individual (centroid size) are produced (Zelditch et al., 2012). The separation between shape and size data into distinct vectors allowed us to latter test for a correlation between the two (i.e., test the presence of allometry; Zelditch et al., 2012). We used thin‐plate spline deformation grids to plot the variation in cranium shape between individuals and the mean shape using the “plotRefToTarget” function of the *geomorph* package (Adams & Otárola‐Castillo, 2013) (Figure 2b).

We performed a principal component analysis (PCA) using the 16 shape vectors from the GPA, without the centroid size (i.e., estimate of size) to assess whether individuals form distinct shape cluster according to the population (urban and non‐urban). This analysis was performed on the covariance matrix of the procrustes shape variables from all individuals, using the “prcomp” function of the R software. The PCA was plotted using ggplot2 for R (Wickham, 2009). We used thin‐plate spline deformation grids to plot the variation in cranium shape between the individuals on the extremes of the main shape axis (PC1) and the mean shape of all individuals. The choice of PC1 as the main shape axis was based on the broken stick method (Jackson, 1993). We tested for differences in cranium shape between urban and non‐urban populations co‐occurring with and without other fish species using the shape vectors from the GPA through a procrustes linear model, with the “procD.lm” function of the *geomorph* package. This function uses a multivariate technique, where the terms in the model are statistically assessed using the procrustes distances among individuals (i.e., the sum of the squared distances between corresponding landmarks after individuals have been centered and rotated) (Adams et al., 2019). The procrustes distances are used as a measure of sum of squares, which are then evaluated through permutation to obtain a *p*‐value (Anderson, 2001; Goodall, 1991).

We used a two‐block partial least squares analysis to test the allometric relationship between cranium shape and centroid size, which is the geometric estimate of body size. The two‐block partial least squares analysis describes the correlation between two blocks of variables by finding the linear vectors within each block that express the greatest covariance between the blocks (Zelditch et al., 2012). For this, the multidimensional block of shape variables from the GPA was reduced to a linear vector of shape represented by partial least squares scores (PLS scores) and correlated with centroid size. This analysis was performed using the “two.b.pls“ function of the *geomorph* package (Adams & Otárola‐Castillo, 2013). Two‐block partial least squares analyses were also used to test the relationship between cranium shape versus individual specialization (WIC/TNW) and cranium shape versus diet (proportion of chironomids consumed, PC). The distribution of PLS scores for each population and the correlation between the PLS scores describing cranium shape, centroid size, individual specialization (WIC/TNW), and diet of each individual were plotted using the *ggplot2* package for R.

### 2.4 Testing the effect of population density and food availability to individual specialization and trophic morphology

Because population density interacts with food availability, we have assessed its effect on individual specialization. Density and food availability estimates were obtained from Marques et al. (2020), and are compiled in the appendix (Table A1). We built linear mixed‐effect models (LMM) for urban and non‐urban populations separately because the environmental drivers of diet and feeding morphology can widely vary between these environments. We built models using the individual specialization estimate (WIC/TNW) per reach as a response variable and guppy density (GD, ind/m^2^), chironomid biomass (mg/m^2^), fish biodiversity (guppy only or guppy + other fish, BIO), and guppy length (SL, mm) as fixed factors. We used reach identity (RI) within year (YR) as a random factor. Model = WIC/TNW~GD + CB + BIO + SL, and random = ~1|YR/RI. We included fish biodiversity in all models because the presence of predators and competitors can also affect guppy diet (Zandonà et al., 2011). We included body length because body size can affect guppy diet (Zandonà et al., 2015). We included reach within year as random factor because spatial and temporal variation in resources availability could affect individual's diet.

**FIGURE A2:**
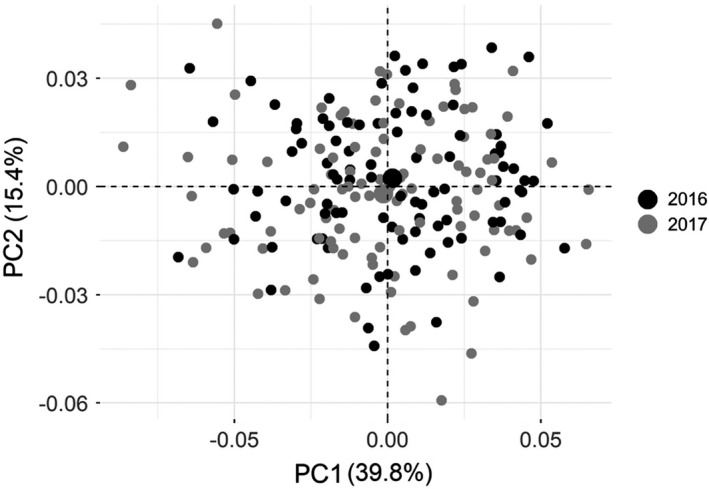
Principal component analysis (PCA) of cranium shape by year. Cranium shape for each individual was obtained from a set of eight anatomical landmarks. The landmarks were converted into 16 shape variables following a generalized procrustes analysis. The 16 vectors of shape were used in the PCA analysis. Each point represents the cranium shape of each individual in both urban and non‐urban populations in 2016 (black circle) and 2017 (grey circle). Large symbols represent the mean of each year

Then, we built procrustes LMM, which uses the cranium shape variables of each individual (16 vectors of shape from the GPA analysis) as the response variable, following the same statistical technique as the procrustes linear model detailed previously. We used the same fixed effects as before but now replacing body length (SL) for centroid size (CS), which is how body size information is recorded after procrustes analysis. We nested reach (RI) as a random effect. We included fish biodiversity in these models because the presence of other fish species such as predators can potentially affect guppy morphology (Torres‐Dowdall et al., 2012). Sampling year (YR) was not included in this model because *a priori* analyses showed that cranium shape was not related to year (Figure A2 in Appendix). The models for cranium shape were fitted using the “procD.lm” function from the *geomorph* package for R (Adams & Otárola‐Castillo, 2013). One sampling reach/year (FLOR2017) was removed from all models because of missing density estimates (Table A1 in Appendix).

## 3 RESULTS

### 3.1 Guppy diet and individual specialization

Urbanization did not affect individual specialization metrics. We found no significant effect of urbanization on WIC (11.8 ± 4.5 and 8.2 ± 2.3, respectively), BIC (3.9 ± 1.1 and 9.4 ± 3.7, respectively), TNW (15.7 ± 5.6 and 17.6 ± 4, respectively), or WIC/TNW (0.7 ± 0.04 and 0.5 ± 0.09, respectively) irrespective of guppy length and year (Table A2 in Appendix). We found no evidence of significant individual specialization (WIC/TNW Monte‐Carlo p‐value) in urban or non‐urban populations (Figure A4, Table A3 in Appendix).

### 3.2 Trophic morphology

**FIGURE 3:**
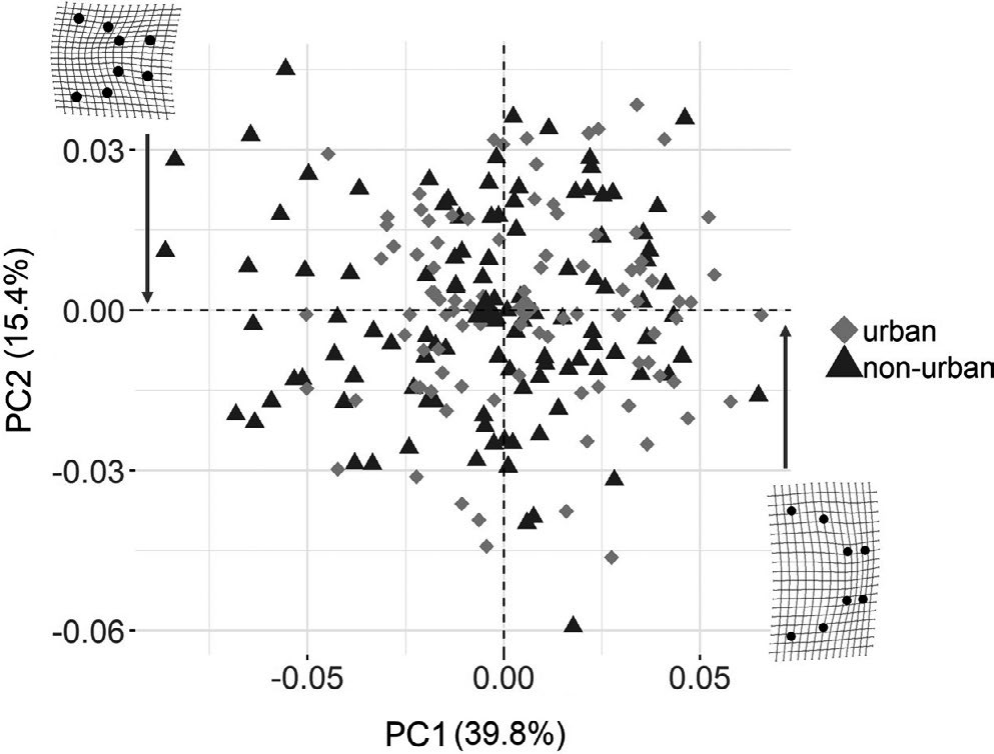
Principal component analysis (PCA) of cranium shape. Cranium shape for each individual was obtained from a set of eight anatomical landmarks. The landmarks were converted into 16 shape variables following a generalized procrustes analysis. The 16 vectors of shape were used in the PCA analysis. Each point represents data from an individual in urban (gray diamonds) and non‐urban (black triangles) populations. Large symbols represent the mean cranium shape of each population. The shape variation is shown as deformation grids of the difference between the specimens on the extremes of the main shape axis (PC1). Individuals toward the left side of PC1 have more narrow/long cranium shape, while individuals on the right have more wide/short cranium shape. The effect of body size is removed from this analysis. Deformation grids were plotted with 1.5× magnification to facilitate visualization of shape differences. Data were combined across years (2016–2017) and across biodiversity treatments (streams where guppies are the only fish species or streams where guppies co‐occur with other fish)

**FIGURE 4:**
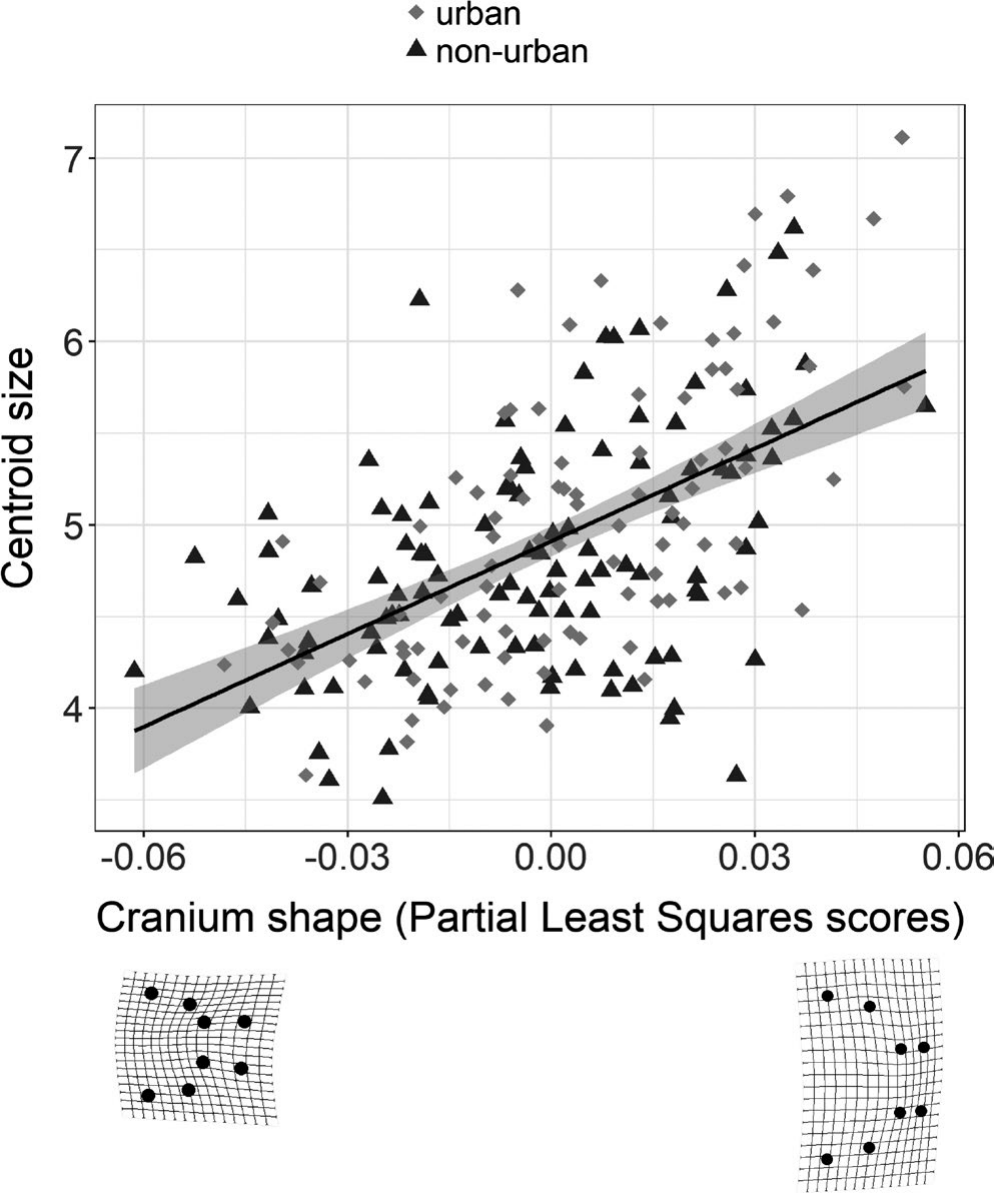
The relationship between cranium shape and body size (i.e., centroid size). Cranium shape for each individual was obtained from a set of eight anatomical landmarks. The landmarks were converted into 16 shape variables following a generalized procrustes analysis. The 16 vectors of shape were projected into a singular vector represented by the partial least square scores (PLS scores) that describe the cranium shape of each individual and were correlated with the centroid size which is a measure of body size used in shape analysis. Each symbol represents one individual in urban (gray diamonds) and non‐urban (black triangles) populations. Lower panels show the shape deformations grids based on the difference between the mean shape of all individuals and the specimens with minimum (left)/maximum (right) partial least square scores. Deformation grids were magnified 1.5× to facilitate visualization of shape differences. Data were combined across years (2016–2017) and across biodiversity treatments (streams where guppies are the only fish species or streams where guppies co‐occur with other fish)

**FIGURE 5:**
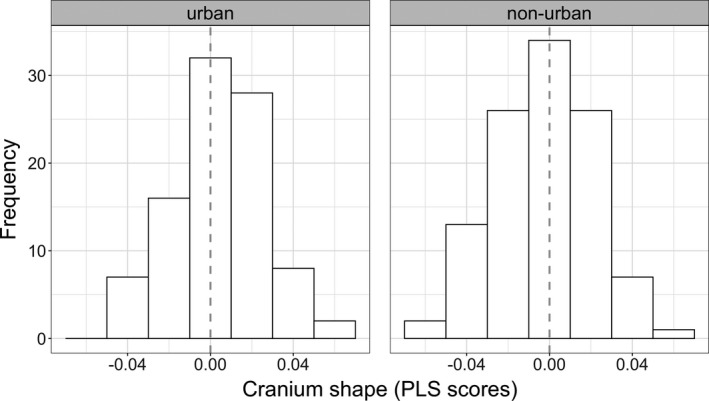
Distribution of the partial least squares scores obtained from 16 vectors of shape that were projected into a singular vector (PLS scores) that describe cranium shape in urban and non‐urban populations. Dashed line marks the zero value for ease of the interpretation. Urban populations have increased frequency of guppies toward positive scores (shorter/wider cranium). Data were combined across years (2016–2017) and across biodiversity treatments (streams where guppies are the only fish species or streams where guppies co‐occur with other fish)

Urbanization had a significant effect on trophic morphology. A PCA analysis of shape showed a slight tendency for urban guppies to cluster on the right of PC1 toward a shorter–wider cranium shape, while non‐urban guppies showed a tendency to cluster toward the left side of PC1 (Figure 3). The PCA eigenvalues and variance explained for each axis can be found in the appendix (Table A4). The procrustes linear model test confirmed shape difference between urban and non‐urban guppies (*F*_1,201_ = 5.4, *p *= .001) and no effect of fish biodiversity. An allometric effect exists because larger individuals had shorter/wider crania (partial least squares correlation coefficient = 0.55, *p *= .001) (Figure 4). Urban populations had high frequency of individuals with high partial least squares scores than non‐urban populations, which suggest that urban populations have more guppies with shorter/wider cranium (Figure 5).

**FIGURE 6:**
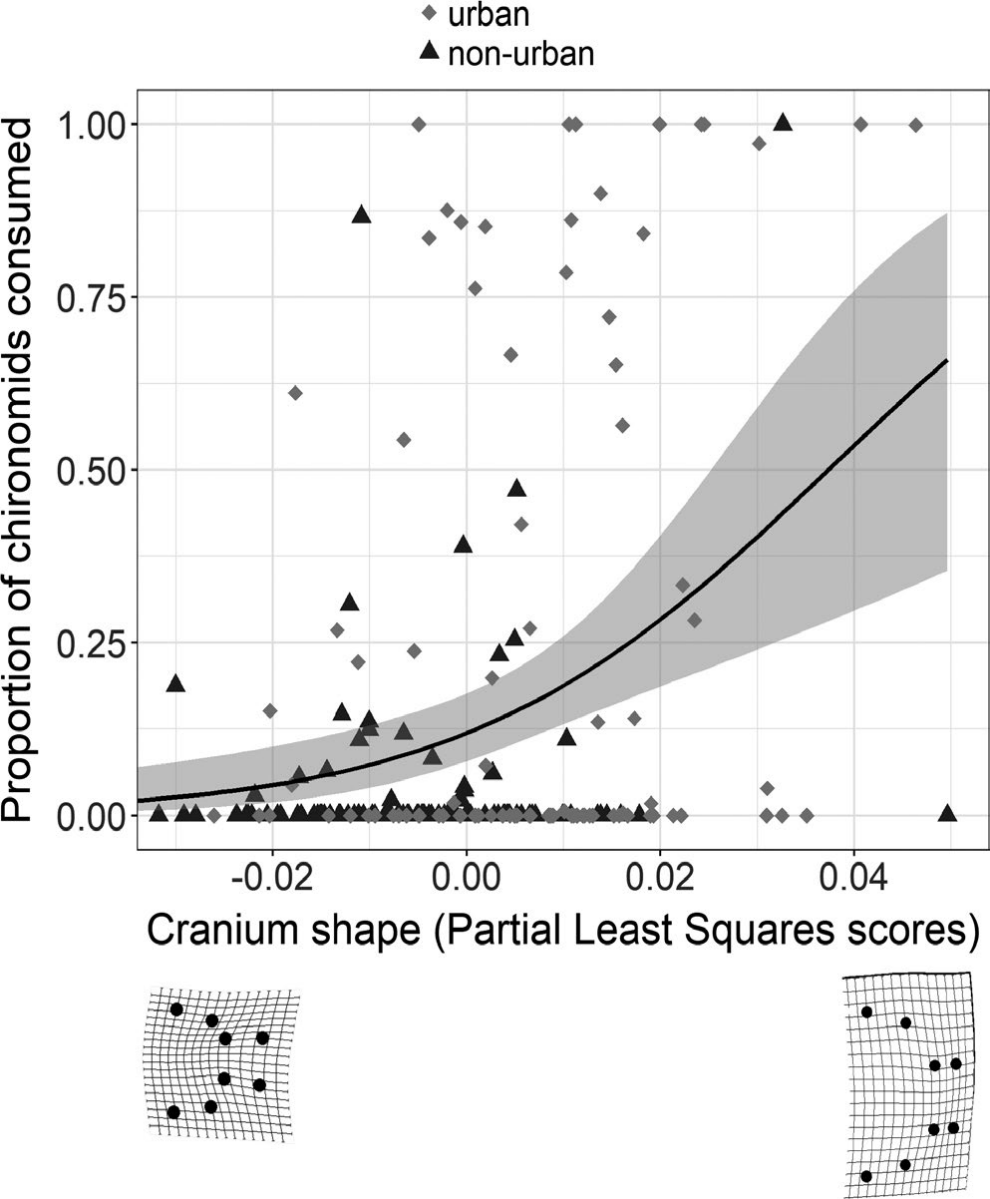
The relationship between cranium shape and the consumption of chironomids (midge larvae) obtained from the two‐block partial least squares analyses. Cranium shape for each individual was obtained from a set of eight anatomical landmarks. The landmarks were converted into 16 shape variables following a generalized procrustes analysis. The 16 vectors of shape were projected into a singular vector represented by the partial least square scores (PLS scores) that describe the cranium shape of each individual and were correlated with the proportion of chironomids consumed. The proportion of chironomids was estimated considering the amount (area of a gridded slide, mm) of all the food items consumed. Each symbol represents one individual in urban (gray diamonds) and non‐urban (black triangles) populations. Lower panels show the shape deformations grids based on the difference between the mean shape of all individuals and the specimens with minimum (left)/maximum (right) partial least square scores. Deformation grids were magnified 1.5× to facilitate visualization of shape differences. Data were combined across years (2016–2017) and across biodiversity treatments (streams where guppies are the only fish species or streams where guppies co‐occur with other fish)

**FIGURE A3:**
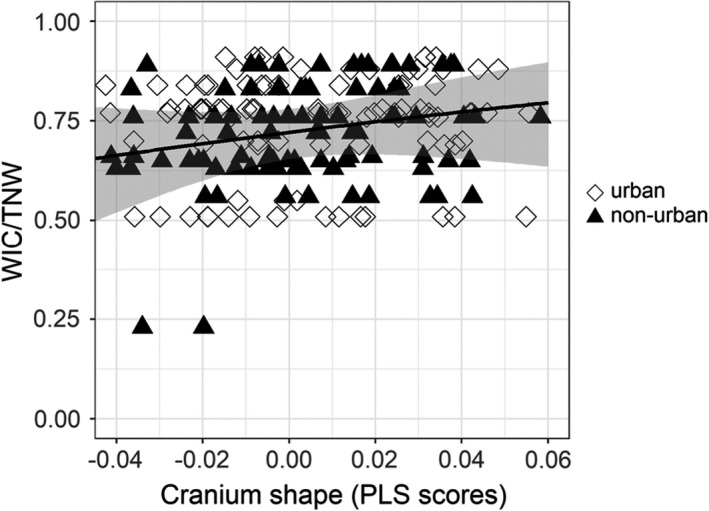
Relationship between feeding morphology (cranium shape) and individual specialization (WIC/TNW). Cranium shape for each individual was obtained from a set of eight anatomical landmarks. The landmarks were converted into 16 shape variables following a generalized procrustes analysis. The 16 vectors of shape were projected into a singular vector represented by the partial least square scores (PLS scores) that describe the cranium shape of each individual were correlated with the centroid size which is a measure of body size used in shape analysis. Each symbol represents one individual in urban (gray diamonds) and non‐urban (black triangles) populations

**FIGURE A4:**
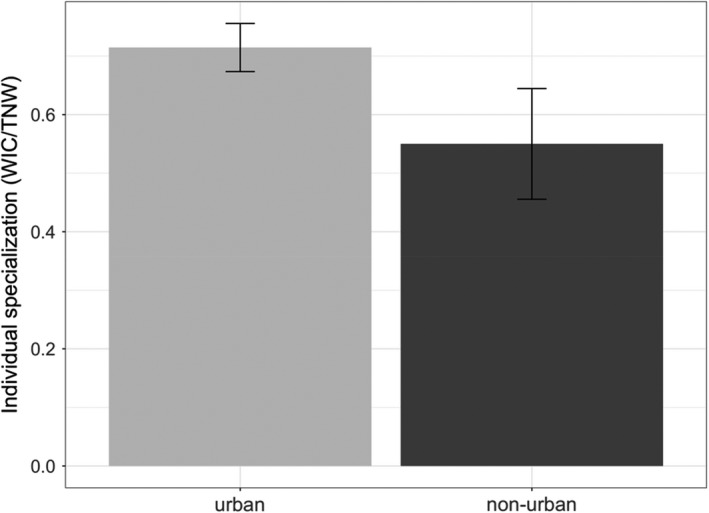
Individual specialization index (WIC/TNW) for urban (light gray) and non‐urban (dark gray) guppy populations. This index is estimated based on the ratio of the total niche width of the population (TNW) and individuals niche width (WIC). Black bars show the standard error of the mean

There was no relationship between cranium morphology and individual specialization metrics (WIC/TNW) (Figure A3 in Appendix). However, individuals with shorter and wider crania tended to consume more chironomids (partial least squares scores correlation coefficient = .33, *p *= .002) (Figure 6).

### 3.3 The effect of food availability and population density on individual specialization and trophic morphology

The LMM models show no effect of food availability or guppy density on WIC/TNW, irrespective of fish biodiversity (Table A5 in Appendix). However, the procrustes linear model showed that chironomid biomass is related to shorter/wider crania in both urban and non‐urban guppies (*F*_6,82_ = 1.8, *p *< .01 and *F*_6,96_ = 4.8, *p *< .01, respectively) (Table 1). Guppy density had an effect, favoring shorter/wider crania in urban guppies (*F*_1,82_ = 3.2, *p *< .01) but had no effect on non‐urban guppies. While fish diversity showed no effect on urban guppies, it reduced cranium length and width of non‐urban guppies (*F*_3,96_ = 2.1, *p *< .01) (Table 1).

**TABLE 1:** Procrustes linear models testing the effect of density and food availability on feeding morphology (cranium shape)

Fixed factors	Urban populations					Non‐urban populations				
	*df*	SS	MS	*F*	*p*‐value	*df*	SS	MS	*F*	*p*‐value
CS	1	0.021	0.021	12.1	.001	1	0.015	0.015	8.4	.001
CB	6	0.019	0.003	1.8	.001	6	0.051	0.008	4.8	.001
BIO	2	0.004	0.002	1.2	.105	3	0.011	0.004	2.1	.002
GD	1	0.005	0.005	3.2	.003	2	0.003	0.002	1.0	.187
residuals	82	0.143	0.002			96	0.170	0.002		

Note

Urban and non‐urban populations were modeled separately. Each model assessed the amount of shape variation attributed to centroid size (variation on shape attributed to body size, CS), chironomid biomass (CB, mg/m^2^), fish biodiversity (BIO, guppy‐only and guppy co‐occurring with other fish species), and guppy density (ind/m^2^). In both models, reach identity was included as a nested random effect (not shown). Where, SS = sum of squares and MS = mean square.

## 4 DISCUSSION

Most urban dwellers have a generalist strategy which allows them to thrive the environmental changes imposed by urbanization (Callaghan et al., 2019; Evans et al., 2011; Lowenstein et al., 2019). Such generalist strategy is most often determined based on the mean trait of all the individuals in the population, with no regard to individual variation. Here, we show that guppies are not individual specialists, having a similar generalist diet, and that urbanization does not lead to changes in individual diet specialization despite massive densities. However, there is important variation in individual feeding morphology (cranium shape) allowing urban guppies to feed more on highly nutritious food.

Urbanization relaxes the effect of guppy density on individual specialization. Density dependence is a central concept in ecology which determine important processes that shape population dynamics (Berryman et al., 2002; Murdoch, 1994). Density is predicted to regulate the strength of intraspecific competition because high density reduces the availability of resources per capita (Svanbäck et al., 2015). This regulation is thought to enhance individual specialization in many species (Araújo et al., 2011; Bolnick et al., 2003; Roughgarden, 1974). However, although urban guppies occur under densities up to 26x higher than non‐urban guppies (Marques et al., 2020), we found that both urban and non‐urban populations are composed of individual generalists (both have high WIC\TNW, indicating no individual specialization). The relaxation of the density effect on guppy individual specialization requires further testing, but it is likely mediated by the increased food availability.

Urban streams sampled for this study and elsewhere have low richness of invertebrates, but high biomass of a few tolerant taxa such as chironomids (Marques et al., 2020; Yule et al., 2015). Chironomids are an important food for guppies and other urban dwellers (Ganassin et al., 2019; Kelly et al., 2019). The high availability of chironomids in urban streams likely eases intraspecific competition, allowing individual guppies to have a generalist feeding despite extremely high density. This is the first study to suggest that high food availability can relax the effect of density on individual specialization in urban dwellers. It is likely that explicitly considering the effect of food availability on the trophic ecology of urban generalists can help clarify current contradictions found among studies on the effect of urbanization on individual specialization (Larson et al., 2020; Newsome et al., 2015).

Further investigating the interaction among density, food availability, and competition in urban systems can be important to better understand the success of urban generalists. In the early‐mid stages of the urbanization process, there is a general increase in population density for most species (Shochat et al., 2010). However, as urbanization advances, generalist species are predicted to dominate, reaching increasingly higher populations densities (Shochat et al., 2010). Mechanisms that relax density‐mediated effects on traits, such as the human‐related increase in food subsidies suggested by our data, can help explain why populations of urban generalists can support extremely large densities without collapsing. This can be an important feature that facilitates the success of urban generalists. In the future, laboratory experiments that test for the effect of varying densities on different traits can be valuable to assess how widespread is the relaxation of density‐mediated effects on urban generalists.

Despite the lack of individual specialization in diet, guppies show variation in feeding morphology that affects feeding efficiency. The cranium shape of urban and non‐urban guppies span from long and narrow to short and wide (Figure 3). This variation is affected by body size: larger guppies have even shorter and wider crania. Such allometric effect is more noticeable in urban guppies because they attain larger body sizes than non‐urban guppies (Marques et al., 2020). Urban guppies with shorter and wider cranium feed more on chironomids (Figure 6). Variation in cranium shape is not followed by changes to individual specialization likely because on average guppies tend to feed on chironomids and a short and wide cranium enhances gape size, which increase efficiency for feeding on such food (Palkovacs et al., 2011; Zandonà et al., 2011).

Increased feeding efficiency of urban generalists has also been shown in doves, *Zenaida asiatica* and *Zenaida macroura*, and gray squirrels, *Sciurus carolinensis* (Bowers & Breland, 1996; Shochat, 2004), but the ecological mechanisms are unclear. Studies often relate increased feeding efficiency in urban systems to behavioral changes (Shochat et al., 2010). However, here we show that feeding morphology can also enhance the feeding efficiency of urban generalists. To the best of our knowledge, this is the first time that morphology has been related to feeding efficiency in urban streams. Despite that, a causal link between morphology and feeding efficiency in urban streams still needs to be tested in feeding experiments.

Exposing the drivers of feeding efficiency is fundamental to better understanding the success of generalists in urban systems. Increased feeding efficiency is linked to nutrition which determines survival and reproductive success (Lowe et al., 2016; Pollock et al., 2017). This can facilitate generalists to competitively exclude other species and dominate urban systems (Shochat et al., 2010). Such information can help move urban ecology toward understanding the mechanisms responsible for controlling biodiversity in cities (McDonnell & Hahs, 2013).

## CONFLICT OF INTERESTS

The authors have no conflict of interest to declare.

## AUTHOR CONTRIBUTION

**Piatã Marques:** Conceptualization (equal); Formal analysis (lead); Investigation (lead); Writing – original draft (lead). **Eugenia Zandonà:** Resources (equal); Writing – review & editing (equal). **Rosana Mazzoni:** Resources (equal); Writing – review & editing (equal). **Rana El‐Sabaawi:** Conceptualization (equal); Resources (equal); Supervision (equal); Writing – review & editing (equal).

## Data Availability

Data are available from Scholars Portal Dataverse: https://doi.org/10.5683/SP3/MLPWZB.

## 1

**TABLE A1:** Estimated guppy biomass and chironomid biomass. Estimates are shown for each reach (RI) and origin (urban or non‐urban stream reach)

RI	Origin	YR	BIO	GD (se)	CB (se)
ANC	Urban	2016	GF	49.4 (160)	82.8 (41.4)
ANC	Urban	2017	GF	10.6 (9)	63.8 (21.9)
CAR	Urban	2016	GO	151.6 (0.5)	201.5 (43.9)
CAR	Urban	2017	GO	367.3 (0.3)	8.2 (2.1)
CATO	Urban	2017	GO	47.6 (1)	180.3 (50.9)
ELSU	Urban	2016	GO	24.7 (1)	113.1 (45.9)
ELSU	Urban	2017	GO	44.9 (0.1)	36.2 (45.9)
FLOR	Urban	2016	GF	37.7 (0.4)	26.1 (6.9)
FLOR	Urban	2017	GF	NA	285.7 (6.9)
WPS	Urban	2016	GF	22.3 (4)	416.1 (15.1)
WPS	Urban	2017	GF	28.4 (2)	8.2 (3.5)
CAML	Non‐urban	2016	GF	0.6 (17)	1.9 (0.4)
CAML	Non‐urban	2017	GF	2.3 (16)	40.5 (9.6)
ELLI	Non‐urban	2016	GO	2.6 (4)	23.9 (12.9)
ELLI	Non‐urban	2017	GO	7.5 (0.4)	2.3 (12.9)
JOA	Non‐urban	2016	GO	0.9 (13)	29.5 (4.3)
TNG	Non‐urban	2017	GF	3.6 (10)	12.9 (4.1)
UBA	Non‐urban	2017	GF	1.2 (16)	32.4 (14.7)
WPL	Non‐urban	2016	GO	4.1 (13)	5.7 (1.6)
WPL	Non‐urban	2017	GO	7.2 (109)	20.5 (7.7)

Note

Urban stream reaches are ANC = Antes Comu, CAR = Carioca, CATO = Catonho, ELSU = Eldo sujo, FLOR = Floresta, and WPS = Water Planet Sujo. While non‐urban stream reaches are CAML = Camorim limpo, ELLI = Eldo limpo, JOA = Joana, TNG = Tingui, UBA = Ubatiba, and WPL = Water Planet Limpo. The fish biodiversity (BIO, guppy‐only (GO) or guppy co‐occurring with other fish species (GF)), guppy density (ind/m^2^), and chironomid biomass (CB, mg/m^2^) are compiled from our previous work, Marques et al. (2020). Guppy density was estimated following a depletion method (Carle and Strub 1978) while chironomid biomass was estimated based on mass‐length regressions (Benke et al., 1999). Detailed information on sampling procedures can be found in Marques et al. (2020).

**TABLE A2:** Linear models testing the effect of urbanization on individual specialization

Fixed factors	Value	STE	*df*	*t*‐value	*p*‐value
WIC/TNW					
Intercept	0.255	0.44	10	0.579	.575
SL	0.009	0.02	5	0.522	.624
Origin	0.145	0.11	10	1.363	.202
Year	0.141	0.1	5	1.347	.236
WIC					
Intercept	−25.613	22.05	10	−1.161	.272
SL	1.552	0.93	5	1.675	.155
Origin	0.774	5.31	10	0.146	.887
Year	−1.716	5.26	5	−0.326	.757
TNW					
Intercept	−24.486	29	10	−0.844	.418
SL	1.989	1.22	5	1.635	.163
Origin	−5.372	7.23	10	−0.743	.474
Year	−4.947	6.77	5	−0.73	.498
BIC					
Intercept	3.667	16.56	10	0.221	.829
SL	0.333	0.69	5	0.48	.652
Origin	−6.056	4.09	10	−1.48	.17
Year	−3.471	3.89	5	−0.892	.413

Note

Models were built using each individual specialization metrics (total niche width of the population (TNW)), within individual's diet (WIC), variability in diet between individuals (BIC) and the degree of individual specialization (WIC/TNW)) as a response variable. The average standard length per reach (SL, mm), origin (urban or non‐urban), and year (2016–2017) were used as fixed factors.

**TABLE A3:** Individual specialization metrics per reach (RI) for urban and non‐urban populations (Origin)

RI	Origin	YR	SL (se)	BIO	*N*	WIC	BIC	TNW	WIC/TNW	*p*‐value
ANC	Urban	2016	31 (1.5)	GF	14	25.12	7.19	32.32	0.78	.80
ANC	Urban	2017	23.7 (1.2)	GF	9	5.38	0.75	6.13	0.88	.92
CAR	Urban	2016	23.2 (1.1)	GO	15	2.55	3.86	6.42	0.40	.33
CAR	Urban	2017	27.6 (2.2)	GO	7	3.47	1.15	4.62	0.75	.92
CATO	Urban	2017	23.8 (2.4)	GO	7	4.62	1.84	6.46	0.71	.82
ELSU	Urban	2016	20 (0.8)	GO	6	0.92	0.26	1.18	0.78	.88
ELSU	Urban	2017	23.2 (0.8)	GO	15	13.68	6.78	20.46	0.67	.83
FLOR	Urban	2016	25.2 (1)	GF	11	16.95	6.87	23.82	0.71	.69
FLOR	Urban	2017	23.4 (0.7)	GF	10	6.61	1.40	8.00	0.82	.87
WPS	Urban	2016	26.8 (1)	GF	15	45.82	10.43	56.26	0.81	.92
WPS	Urban	2017	19.5 (1.3)	GF	4	0.40	0.32	0.73	0.55	.43
CAML	Non‐urban	2016	25.7 (0.8)	GF	17	0.61	0.94	1.55	0.39	.12
CAML	Non‐urban	2017	21.7 (1.2)	GF	10	13.10	5.62	18.71	0.70	.60
ELLI	Non‐urban	2016	22.9 (0.7)	GO	11	0.88	33.14	34.02	0.03	.17
ELLI	Non‐urban	2017	18.6 (0.6)	GO	18	16.91	6.60	23.51	0.72	.88
JOA	Non‐urban	2016	20.2 (1)	GO	8	2.38	1.28	3.66	0.65	.56
TNG	Non‐urban	2017	25 (0.9)	GF	17	14.93	2.14	17.07	0.87	.97
UBA	Non‐urban	2017	21.1 (0.9)	GF	13	3.45	0.79	4.24	0.81	.72
WPL	Non‐urban	2016	24.6 (1.1)	GO	14	16.26	12.25	28.50	0.57	.39
WPL	Non‐urban	2017	22.1 (1)	GO	10	5.82	21.64	27.46	0.21	.06

Note

Urban stream reaches are ANC = Antes Comu, CAR = Carioca, CATO = Catonho, ELSU = Eldo sujo, FLOR = Floresta, and WPS = Water Planet Sujo. While non‐urban stream reaches are CAML = Camorim limpo, ELLI = Eldo limpo, JOA = Joana, TNG = Tingui, UBA = Ubatiba, and WPL = Water Planet Limpo. Where: YR = sampling year, SL = mean guppy length (mm), BIO = fish biodiversity (guppy only (GO) or guppy and other fish (GF)), *N* = number of individuals, TNW = total niche width of the population, WIC = variation in resource use within individual's diet, BIC = variation in diet between individuals, and WIC/TNW = the degree of individual specialization. The *p*‐value is generated after a Monte Carlo resampling routine that tests the null hypothesis that all individuals sample equally from the population diet distribution.

**TABLE A4:** Principal component analysis (PCA) loadings for the cranium shape variables obtained from a set of eight anatomical landmarks

PCs	Eig.	Var (%)	C.var (%)
PC1	0.00090	39.8	39.8
PC2	0.00035	15.4	55.2
PC3	0.00023	10.2	65.3
PC4	0.00017	7.7	73.1
PC5	0.00013	5.7	78.8
PC6	0.00012	5.2	83.9
PC7	9.19 × 10^−5^	4.1	88.0
PC8	7.9 × 10^−5^	3.5	91.5
PC9	7.17 × 10^−5^	3.2	94.7
PC10	4.44 × 10^−5^	2.0	96.6
PC11	4.34 × 10^−5^	1.9	98.5
PC12	3.3 × 10^−5^	1.5	100
PC13	9.87 × 10^−32^	4.36 × 10^−27^	100
PC14	8.89 × 10^−32^	3.93 × 10^−27^	100
PC15	2.15 × 10^−33^	9.52 × 10^−29^	100
PC16	9.61 × 10^−34^	4.25 × 10^−29^	100

Note

The landmarks were converted into 16 shape variables following a generalized procrustes analysis. The 16 vectors of shape were used in the PCA analysis. The eigenvalues (Eig), variance (Var), and cumulative variance (C.var) that each principal component (PC1‐16) explain are shown. Data were combined across years (2016–2017) and across biodiversity treatments (streams where guppies are the only fish species or streams where guppies co‐occur with other fish).

**TABLE A5:** Linear models testing the effect of density and food availability on individual specialization

Fixed factors	Urban population				Non‐urban population					
	Coefficient	STE	*df*	*t*‐value	*p*‐value	Coefficient	STE	*df*	*t*‐value	*p*‐value
int	0.271	0.463	4	0.585	.590	2.571	1.517	3	1.694	.189
SL	0.020	0.019	4	1.041	.357	−0.074	0.057	3	−1.296	.286
GO vs. GF	−0.014	0.131	4	−0.109	.918	−0.412	0.242	3	−1.705	.187
CB	−0.0001	0.001	4	−0.307	.774	−0.006	0.009	3	−0.650	.562
GD	−0.0004	0.001	4	−0.654	.548	−0.003	0.053	3	−0.054	.960

Note

Urban and non‐urban populations were modeled separately. Each model assessed how the individual specialization metric WIC/TNW relates to body size (SL, mm), chironomid biomass (CB, mg/m^2^), fish biodiversity (guppy only (GO) and guppy co‐occurring with other fish species (GF)), and guppy density (ind/m^2^). In both models, reach identity was included as a nested random effect (not shown). Where, SS = sum of squares and MS = mean square.
